# Comparison of traumatic brain injury resulting from stair-related falls to falls from standing height—a neurotrauma center cohort

**DOI:** 10.3389/fneur.2025.1599229

**Published:** 2025-07-04

**Authors:** Cathrine Tverdal, Andrew Reiner, Eirik Helseth, Torgeir Hellstrøm, Unn Sollid Manskow, Mads Aarhus, Karoline Skogen, Pål Rønning, Dag Ferner Netteland

**Affiliations:** ^1^Department of Neurosurgery, Oslo University Hospital, Oslo, Norway; ^2^Oslo Centre for Biostatistics and Epidemiology, Institute of Basic Medical Sciences, Faculty of Medicine, University of Oslo, Oslo, Norway; ^3^Faculty of Medicine, Institute of Clinical Medicine, University of Oslo, Oslo, Norway; ^4^Department of Physical Medicine and Rehabilitation, Oslo University Hospital, Oslo, Norway; ^5^Centre for Clinical Documentation and Evaluation (SKDE), Northern Norway Regional Health Authority, Tromsø, Norway; ^6^Department of Health and Care Sciences, Centre for Care Research, UiT- The Arctic University of Norway, Tromsø, Norway; ^7^Department of Radiology, Oslo University Hospital, Oslo, Norway

**Keywords:** traumatic brain injury, epidemiology, falls, stairs, standing height, adults, older

## Abstract

**Background:**

Falls are the leading cause of traumatic brain injury (TBI) in high-income countries, and globally, the incidence of fall-related injuries is projected to rise. In this study, we compare TBI resulting from stair-related falls (SRFs) to falls from standing height (FSH), analyzing their epidemiology and outcomes.

**Methods:**

In a single-center, registry-based cohort study using the Oslo TBI Registry-Neurosurgery (OTBIR-N), we identified adults (≥18 years) admitted to Oslo University Hospital with TBI from either SRFs or FSH between 2015 and 2022. Epidemiology and outcome measures were compared between the two groups, and a multivariate logistic regression model was used to evaluate the adjusted effect of the fall mechanisms on dichotomized functional outcome (Glasgow outcome score (GOS) 1–3 vs. GOS 4–5).

**Results:**

A total of 1,432 patients with a median age of 71 years were included. SRFs represented 25%, while FSH represented 52% of all fall-related TBIs. SRF patients were generally younger and healthier, with a higher frequency of moderate to severe TBI than FSH patients (53% vs. 31%; *p* < 0.001). SRFs also occurred more often during evenings and nights, on weekends, and were more often related to alcohol influence (58% vs. 22%; p < 0.001). Both fall types resulted in unfavorable functional outcomes (GOS 1–3) for a substantial proportion of patients (37% in SRFs and 42% in FSH; *p* = 0.066). When adjusting for covariates in the multivariable logistic regression model, there was a tendency of SRFs being associated with unfavorable outcomes compared to FSH, but the effect was not statistically significant (OR 1.43, 95%CI 0.97–2.12; *p* = 0.073).

**Conclusion:**

SRFs represented a considerable proportion of fall-related TBIs and were associated with poor outcomes in a substantial proportion of patients. Targeted public awareness campaigns addressing the risks associated with staircases, especially when combined with alcohol influence, seem warranted to prevent such injuries.

## 1 Introduction

Falls are the leading cause of traumatic brain injury (TBI) in high-income countries, where they account for approximately half of all TBIs requiring admission to trauma centers (1–6). The World Health Organization describes falls as a growing and under-recognized public health issue and predicts fall-related injuries to rise globally in the coming decades due to factors such as aging populations, increased urbanization, and sedentary lifestyles (7).

Risk factors for falls can be categorized into person-specific (such as advanced age, comorbidities, ethanol influence, polypharmacy, and functional impairments) and environmental causes (such as slippery floors, lack of stair railings, and poor lighting) (7–9). In older adults, falls often occur at home, where stair-related falls account for 11–22% of these incidents (10, 11). Alcohol intoxication has been shown to affect injury patterns with an increased risk of sustaining a TBI after stair-related falls (SRFs) (12, 13). Compared to falls from standing height (FSH), SRFs imply a higher energy trauma and may therefore be hypothesized to result in more severe injury, including TBI (10, 14–16).

Falls represent the leading cause of TBI and account for a considerable proportion of its overall societal impact. Although many individuals recover after sustaining a TBI, a substantial proportion experience reduced function or disability, often compounded by comorbidity and frailty that further complicate recovery (2, 5, 17, 18). A detailed characterization of fall-related TBIs is essential to tailor healthcare systems to meet current needs. This includes optimizing resources and workflows in trauma centers, refining clinical guidelines, strengthening rehabilitation services, and adopting appropriate preventive measures at the societal level.

In this single-center registry-based cohort study, we compare TBI from SRFs to TBI after falls from FSH, detailing their epidemiology and resulting outcomes.

## 2 Materials and methods

### 2.1 Study design and data sources

In this study comparing two common types of falls, we conducted a retrospective, cohort study based on prospectively collected registry data from the Oslo TBI Registry-Neurosurgery (OTBIR-N).

Oslo University Hospital (OUH) serves as the regional neurotrauma center for the Southeast region of Norway, which encompasses a population of approximately 3.1 million and 19 local trauma hospitals covering both urban and rural areas (6). In Norway, trauma care is organized through public hospitals under an equal-access policy and is free of charge. OTBIR-N is a quality control database managed by the Department of Neurosurgery at OUH (6). Prospective registration started in 2015, and data are collected based on available information in electronic medical records. Inclusion criteria for the registry are (i) traumatic brain injury; (ii) cerebral-CT/CTA or cerebral-MRI/MRA with findings of acute trauma (hemorrhage, fracture, traumatic axonal injury, and vascular injury); (iii) admitted to OUH within 7 days post-injury; and (iv) Norwegian social security number.

The current study (24/01340) and OTBIR-N (2016/17569) were approved by the OUH data protection officer.

### 2.2 Patient inclusion

**Figure 1:**
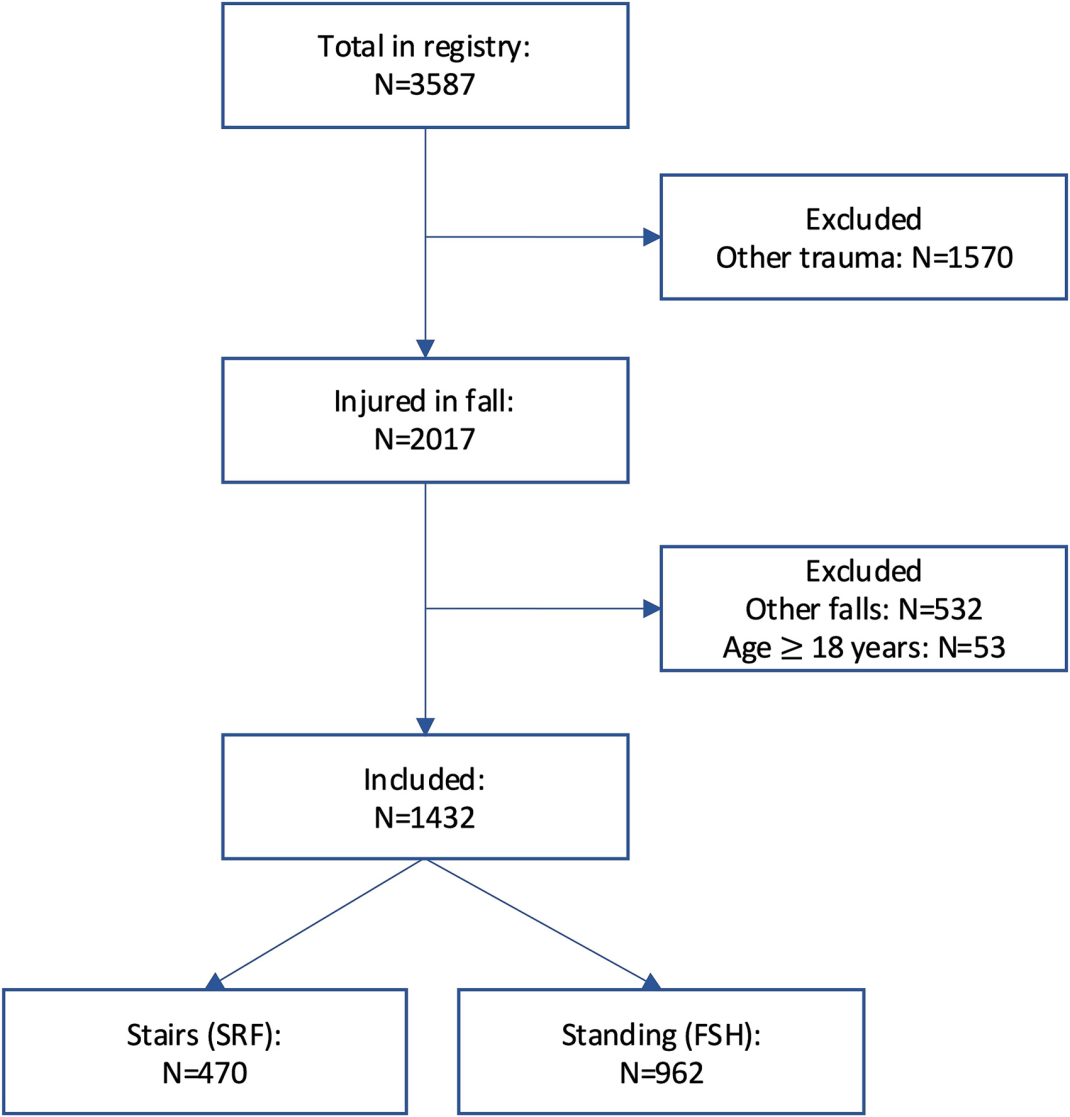
Flow chart of patient inclusion from OTBIR-N.

From OTBIR-N, we identified adults (≥18 years) admitted to OUH between 1 January 2015 and 31 December 2022 who had sustained a TBI confirmed by pathological findings on head CT, either from a stair-related or standing height fall. A fall from standing height is defined in the registry as a fall that begins when a person has his or her feet on the ground, and a stair-related fall is defined as a fall involving one or more steps in a stair. Other categories of falls were not included in the analysis (such as falls from chair/bed, window/balcony, ladder/scaffold, roof, tree, play stand, hillside, and unknown). Patient inclusion is further detailed in the flow chart in Figure 1.

### 2.3 Data collection

All variables were retrieved from OTBIR-N. Demographics and details on pre-injury patient status, including functional living status (home independent/home assisted/institution), American Society of Anesthesiologists Physical Status Classification System (ASA-PS) class (19), and the use of antithrombotics, were gathered. Injury details, including place and time of injury and whether the patient was under the influence of alcohol, were collected. TBI severity indicators included the Glasgow coma scale (GCS) score (20) (recorded as the lowest score prior to intubation or arrival at OUH), pupillary dilatation, and Rotterdam CT score (21). Additional TBI characteristics, including details on types of traumatic intracranial hemorrhages and whether the patient had traumatic injuries other than TBI, were also collected.

Outcome measures include mortality (automatically updated in the registry via a link to the national population registry), Glasgow outcome score (GOS) (22) based on available information in electronic patient records 6 months after trauma, and functional living status 6 months post-trauma in the same format as pre-trauma (home-independent/home-assisted/institution).

### 2.4 Data analysis

The proportion of patients sustaining a TBI from SRFs and FSH to TBI from all fall categories was calculated. Patient characteristics, injury characteristics, TBI severity, and outcome measures were described for the whole cohort and subsequently compared between the SRF group and FSH group using parametric or non-parametric tests as appropriate. Heatmaps were used to describe temporal trends in the incidence of the fall mechanisms, and the proportion of patients who were under the influence of alcohol during the fall was explored and compared between the two groups. UpSet plots were used to describe and compare patterns of TBI subtypes. To evaluate whether the type of fall had an effect on functional outcome, we used a logistic regression model with dichotomized GOS (GOS 1–3 vs. GOS 4–5) as the dependent variable. Age, sex, ASA-PS class, functional living status pre-trauma, GCS, Rotterdam CT score, presence of pupillary dilatation, isolated TBI vs. multitrauma, alcohol influence during the fall, and use of antithrombotics were included as clinically relevant independent variables. Finally, Sankey diagrams were used to illustrate the change in functional living status from pre-trauma to 6 months post-trauma. Statistical analyses were performed using IBM SPSS Statistics version 29, Stata (StataCorp. 2023. Stata Statistical Software: Release 18. College Station, TX: StataCorp LLC.), or R version 4.4.0, and a *p*-value of <0.05 was considered significant.

## 3 Results

**Figure 2:**
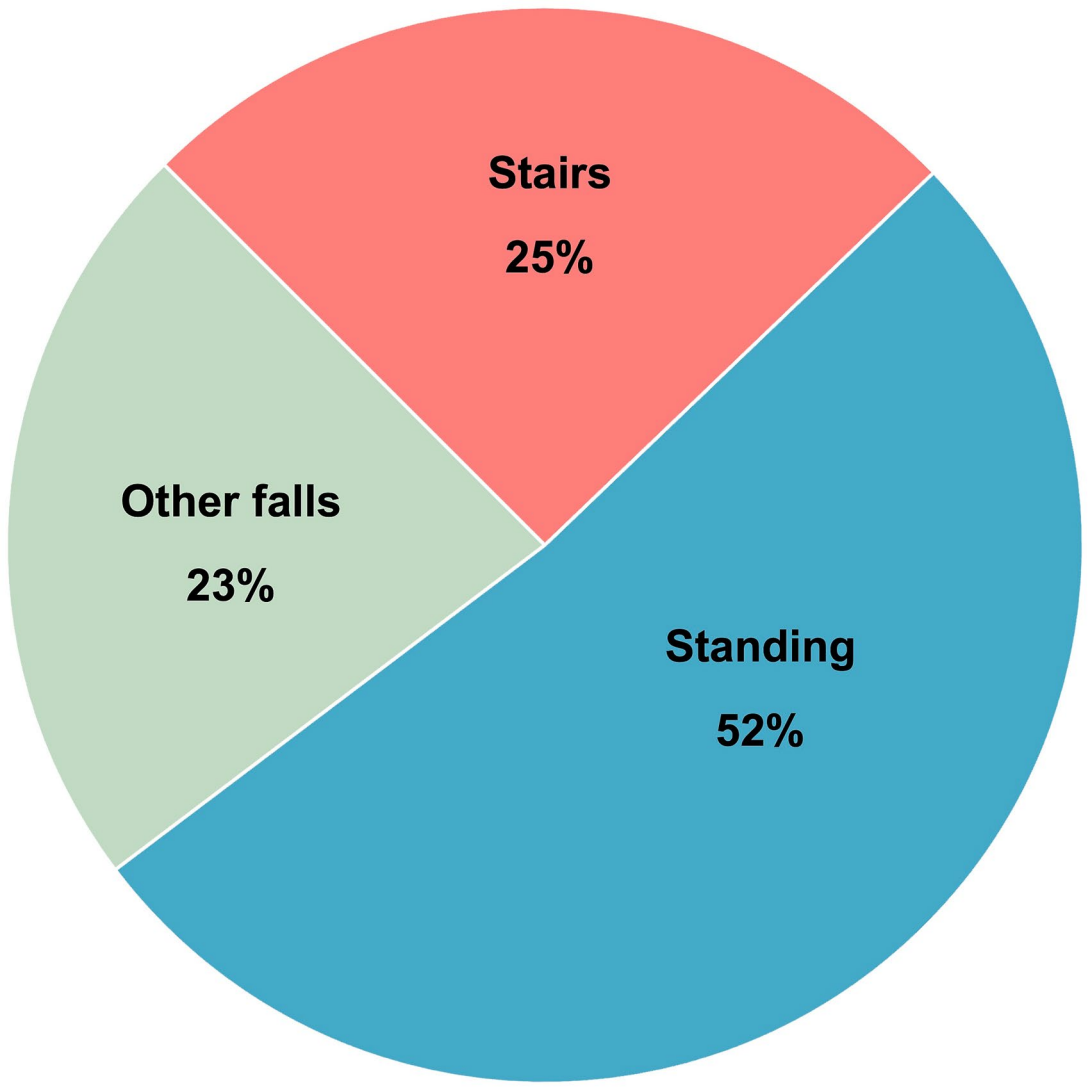
Distribution of fall mechanisms in percent of total in OTBIR-N (*N* = 2017).

A total of 1,432 patients were included in the study: 470 patients in the SRF group and 962 patients in the FSH group. These fall mechanisms represented 25 and 52% of all fall-related TBIs in the OTBIR-N in the time period (Figure 2).

### 3.1 Patient characteristics

Overall, the median age was 71 years (interquartile range (IQR) 58–81 years); 825/1432 (57%) were male. Patients in the SRF group were younger, had fewer comorbidities, lived more independently, and used less anti-thrombotics compared to patients in the FSH group. Patient characteristics for the whole cohort and the two different fall groups are presented in detail in Table 1.

**Table 1:** Patient characteristics.

	All (*n* = 1,432)	SRF (*n* = 470)	FSH (*n* = 962)	*p*-value
Age in years, median (IQR)	71 (58–81)	66 (54–76)	73 (60–83)	<0.001
Age groups				<0.001
18–39	113 (8)	43 (9)	70 (7)	
40–59	270 (19)	106 (23)	164 (17)	
60–79	655 (46)	237 (50)	418 (44)	
80+	394 (28)	84 (18)	310 (32)	
Male	825 (57)	280 (60)	545 (57)	0.29
Living status				<0.001
Home independent	1,102 (77)	416 (89)	686 (71)	
Home assisted	262 (18)	48 (10)	214 (22)	
Nursing home/Other/unknown	68 (5)	6 (1)	62 (6)	
Preinjury ASA				<0.001
1	250 (18)	120 (26)	130 (14)	
2	411 (29)	144 (31)	267 (28)	
3	731 (51)	201 (43)	530 (55)	
4	40 (3)	5 (1)	35 (4)	
Antithrombotic medication:				<0.001
None	785 (55)	319 (68)	466 (48)	
Single platelet inhibitor	335 (23)	91 (19)	244 (25)	
Anticoagulation	241 (17)	53 (11)	188 (20)	
Combination	71 (5)	7 (2)	64 (7)	
Place of injury				<0.001
Private home	741 (52)	299 (64)	442 (46)	
Other	691 (48)	171 (36)	464 (54)	

Data are *n* (%) or median (IQR). p-values are for comparison between the falling down the stairs group and falling from standing height group. FSH, fall from standing height; SRF, stairs-related fall.

### 3.2 Temporal patterns of injury and alcohol influence

**Figure 3:**
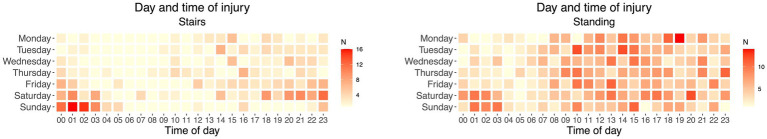
Heat maps visualize the time and day of injury in the two groups. The x-axis denotes the time of day, ranging from 00:00 to 23:59 h, segmented into hourly intervals. The y-axis represents the days of the week, starting from Monday (top) to Sunday (bottom). The color represents the frequency within the time interval, with white colors representing fewer cases and red colors representing higher numbers.

The heat maps (Figure 3) demonstrate an apparent difference in the temporal patterns of injury between the two groups, with SRFs showing a clear preponderance toward evenings and nighttime during the weekends. In contrast, FSH was more evenly distributed with regard to time of day and weekday. Consistent with this, patients in the SRF group were considerably more often under the influence of alcohol compared to patients in the FSH group (274/470 (58%) vs. 213/962 (22%); *p* < 0.001).

**Figure 4:**
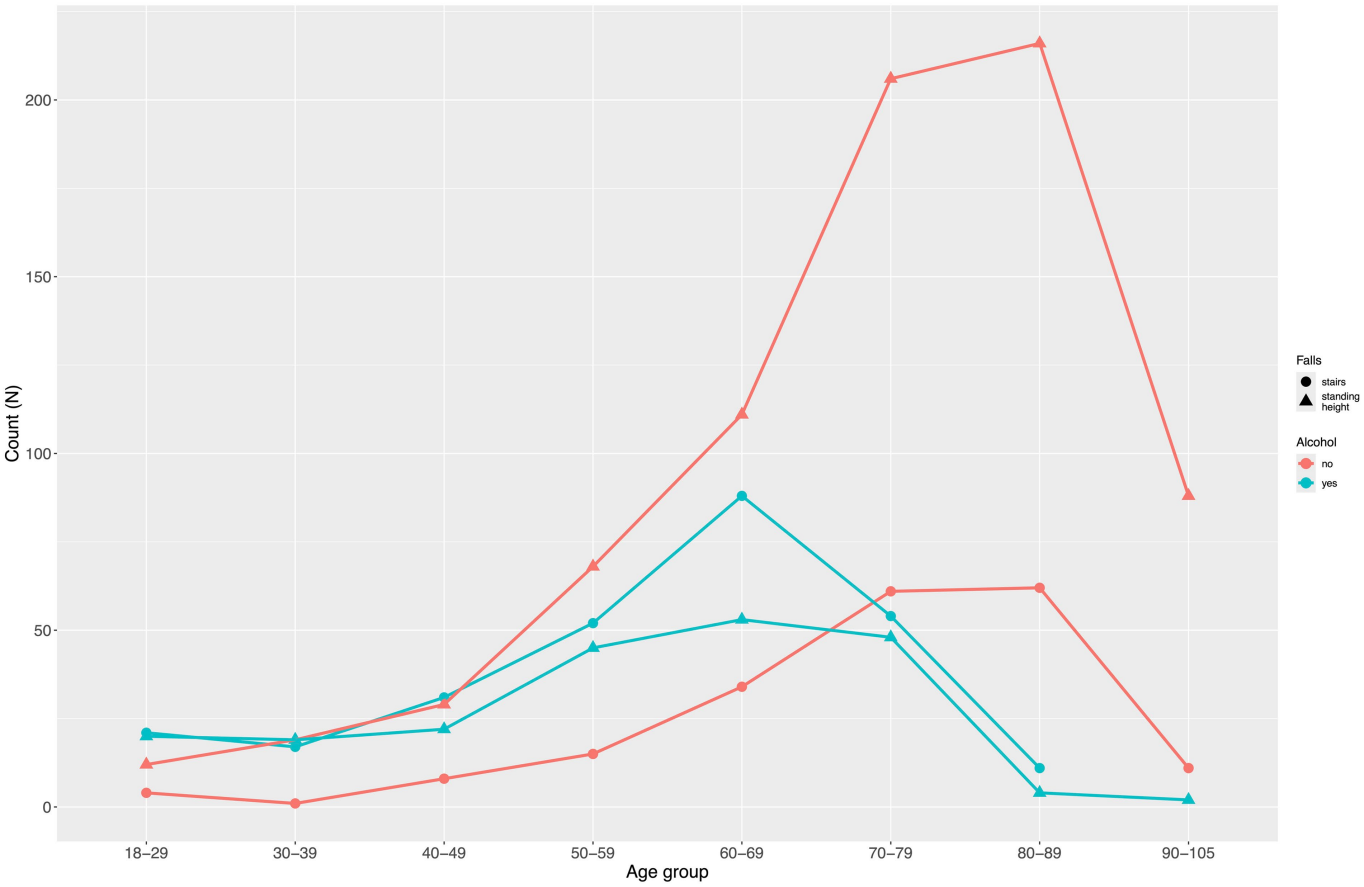
Distribution of patients by age groups, type of fall, and alcohol influence during the fall.

In both groups, the absolute number of falls under alcohol influence peaked in the age group 60–69 years. However, the ratio of patients under the influence of alcohol to those who were not remained highest among those aged <50 years (Figure 4).

### 3.3 TBI severity and lesion types

When comparing the two groups, SRFs had more severe TBIs, with 53% sustaining a moderate or severe TBI (GCS 3–12) as compared to 31% in the FSH group (*p* < 0.001). The Rotterdam CT score also indicated more severe radiological TBI in the former group. Additionally, patients in the SRF group more often demonstrated extracranial injuries (232/470 (49%) vs. 207/962 (22%); p < 0.001), were more often admitted to the intensive care unit (ICU), and more frequently had intracranial pressure (ICP) monitored. TBI severity and characteristics are shown in Table 2.

**Table 2:** Injury characteristics, acute treatment, and discharge.

	All (*n* = 1,432)	SRF (*n* = 470)	FSH (*n* = 962)	*p*-value
Glasgow coma scale score				<0.001
GCS 13–15 (mild)	886 (62)	219 (47)	667 (69)	
GCS 9–12 (moderate)	240 (17)	94 (20)	146 (15)	
GCS 3–8 (severe)	306 (21)	157 (33)	149 (16)	
Rotterdam CT score				<0.001
1–2	449 (31)	136 (29)	313 (33)	
3–4	844 (59)	269 (57)	575 (60)	
5–6	139 (10)	65 (14)	74 (8)	
Pupillary dilatation				0.097
None	1,336 (93)	432 (92)	904 (94)	
Unilateral	57 (4)	21 (5)	36 (4)	
Bilateral	35 (2)	17 (4)	18 (2)	
Unknown	4 (<1)	0	4 (<1)	
Neurosurgical evacuation of mass lesion				
Any mass lesion type	176 (12)	62 (13)	114 (12)	0.468
Epidural hematoma	33 (2)	13 (3)	20 (2)	0.416
Contusion	32 (2)	14 (3)	18 (2)	0.183
Subdural hematoma	134 (9)	49 (10)	85 (9)	0.332
ICP monitor	186 (13)	97 (21)	89 (9)	<0.001
ICU admission	821 (57)	328 (70)	493 (51)	<0.001
Multiple trauma	439 (31)	232 (49)	207 (22)	<0.001
In-hospital mortality	138 (10)	51 (11)	87 (9)	0.276
Discharge destination				<0.001
Home	448 (35)	123 (29)	325 (37)	
Local hospital	550 (43)	202 (48)	348 (40)	
Specialized rehabilitation	136 (11)	66 (16)	70 (8)	
Nursing home	137 (11)	24 (6)	113 (13)	
Other	23 (2)	4 (1)	19 (2)	

Data are *n* (%). *p*-values are for comparison between the stair-related fall group and the standing height fall group. CT, computed tomography; FSH, fall from standing height; ICP, intracranial pressure; ICU, intensive care unit; SRF, stairs-related fall.

**Figure 5:**
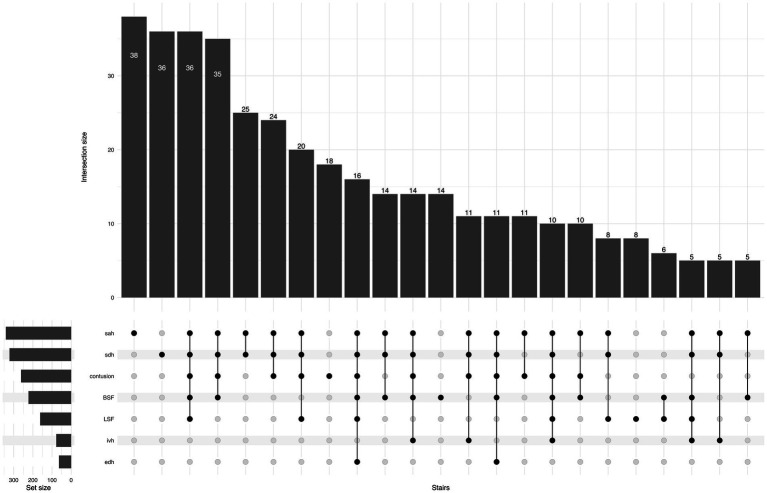
UpSet plot shows traumatic lesions on primary head CT for the SRF group, the number of patients in each lesion/combination is shown at the top of each bar. For ease of reading, the tail in the plot is cut when the number of cases is less than five patients. BSF, basilar skull fracture; EDH, epidural hematoma; IVH, intraventricular hemorrhage; LSF, linear skull fracture; SAH, subarachnoid hemorrhage; SDH, subdural hematoma.

**Figure 6:**
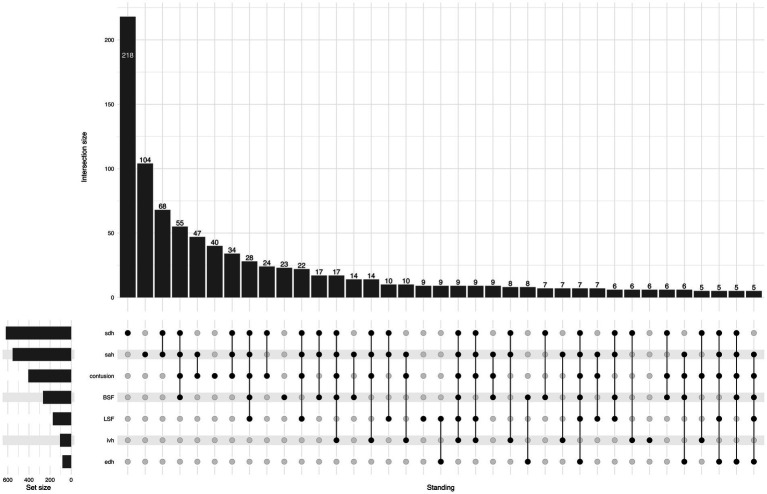
UpSet plot shows traumatic lesions on primary head CT for the FSH group, the number of patients in each lesion/combination is shown at the top of each bar. For ease of reading, the tail in the plot is cut when the number of cases is less than five patients. BSF, basilar skull fracture; EDH, epidural hematoma; IVH, intraventricular hemorrhage; LSF, linear skull fracture; SAH, subarachnoid hemorrhage; SDH, subdural hematoma.

Regarding TBI lesion types, isolated acute subdural hematoma (ASDH) or traumatic subarachnoid hemorrhage (SAH) were the most frequent lesions in both groups. In the SRF group, 26% had an isolated lesion type, while in the FSH group, the corresponding proportion was 41%. Conversely, multiple concomitant lesions were more common for SRFs compared to FSH (73% vs. 58%; *p* < 0.001). The UpSet plot in Figures 5, 6 illustrates the distribution of CT findings in the two groups.

### 3.4 Outcome at 6 months

At 6 months, GOS was available from 1353/1432 (94%) of the included patients (441/470 (94%) in the SRF group and 912/962 (95%) in the FSH group).

**Figure 7:**
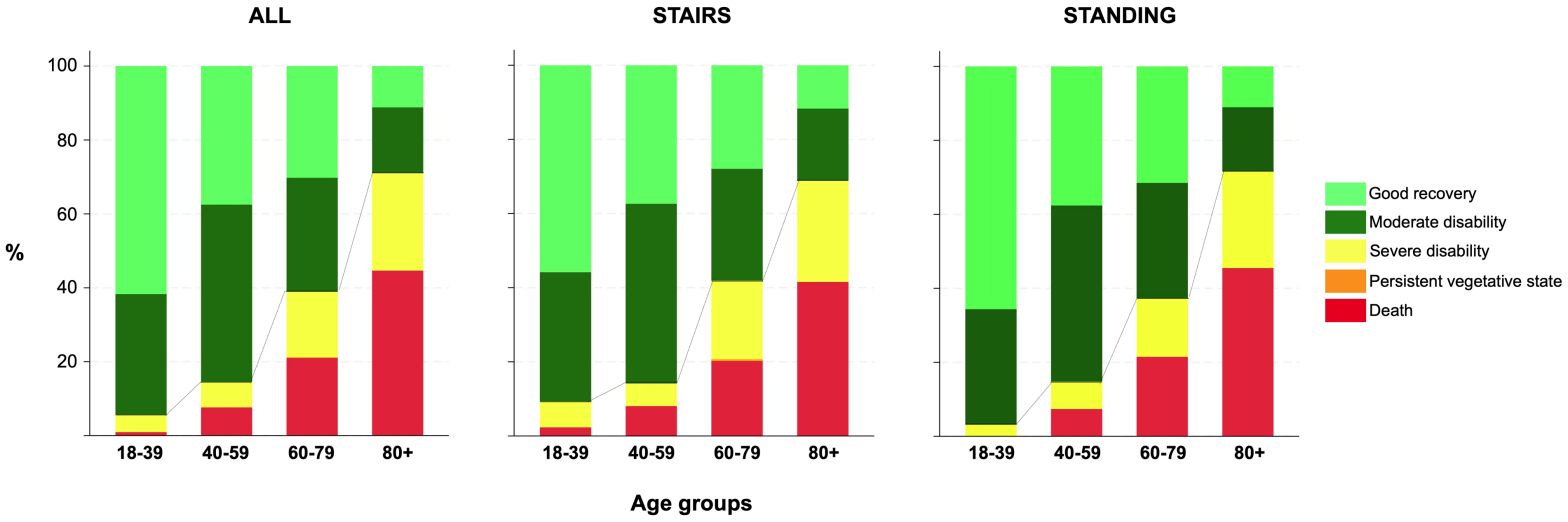
Bar charts for 6-month GOS by age group for all, and the two groups. Line represents proportions of dichotomized GOS and mortality.

For the whole cohort, 551/1353 (41%) had an unfavorable outcome (GOS 1–3) at 6 months, and mortality was 319/1432 (22%). In the SRF group, 164/441 (37%) had an unfavorable outcome compared to 387/912 (42%) in the FSH group (*p* = 0.066). Six-month mortality was 86/470 (18%) in the SRF group and 2033/962 (24%) in the FSH (*p* = 0.012). GOS scores at 6 months are shown in Figure 7.

**Figure 8:**
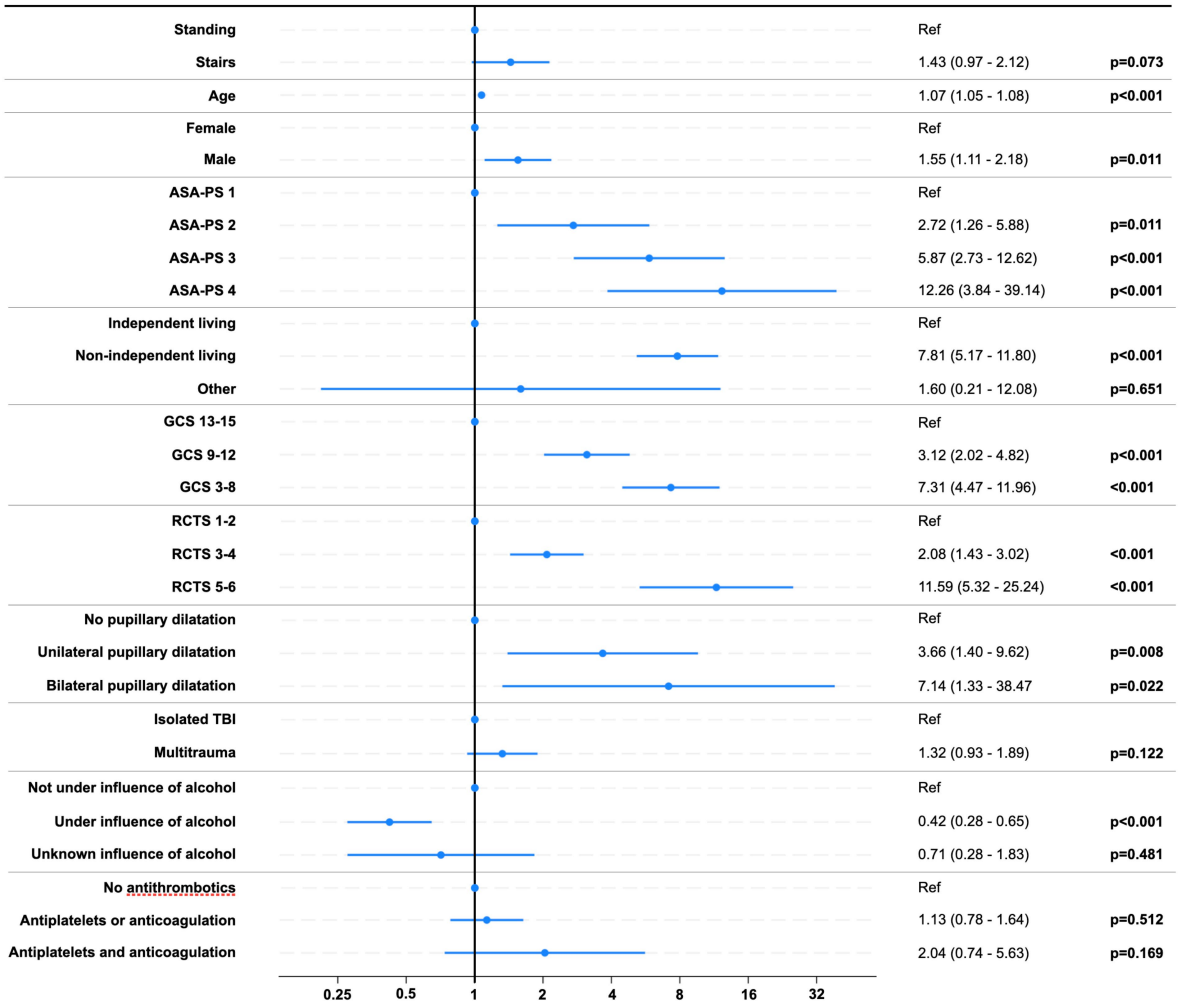
Risk of unfavorable outcome (GOS 1–3). Forest plot of odds ratios with 95% CIs. Abbreviations: ASA-PS, American Society of Anesthesiologists Physical Status Classification System; GCS, Glasgow coma scale score; RCTS, Rotterdam CT score.

When adjusting for covariates in the multivariable logistic regression model for unfavorable outcomes (GOS 1–3), the effect of stair-related fall did not reach statistical significance (OR 1.43, 95%, CI 0.97–2.12; *p* = 0.073). Covariates and results from the regression model are shown in Figure 8.

**Figure 9:**
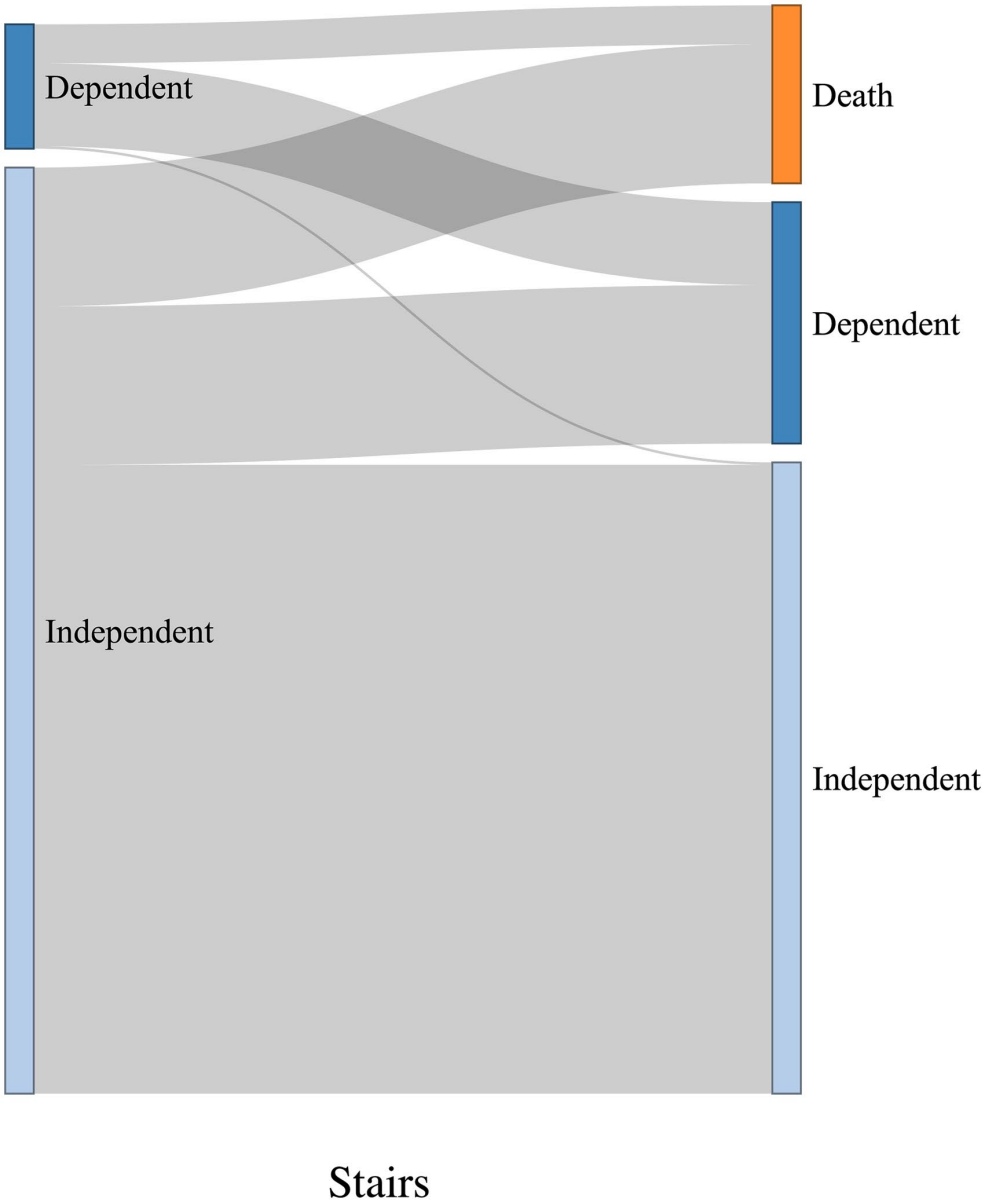
Visualization of change in living status between pre-trauma and 6-month post-trauma for the SRF group. Left side of the diagrams represents pre-trauma, and the right side represents 6-month post-trauma. The height of horizontal columns corresponds to the proportion of patients.

**Figure 10:**
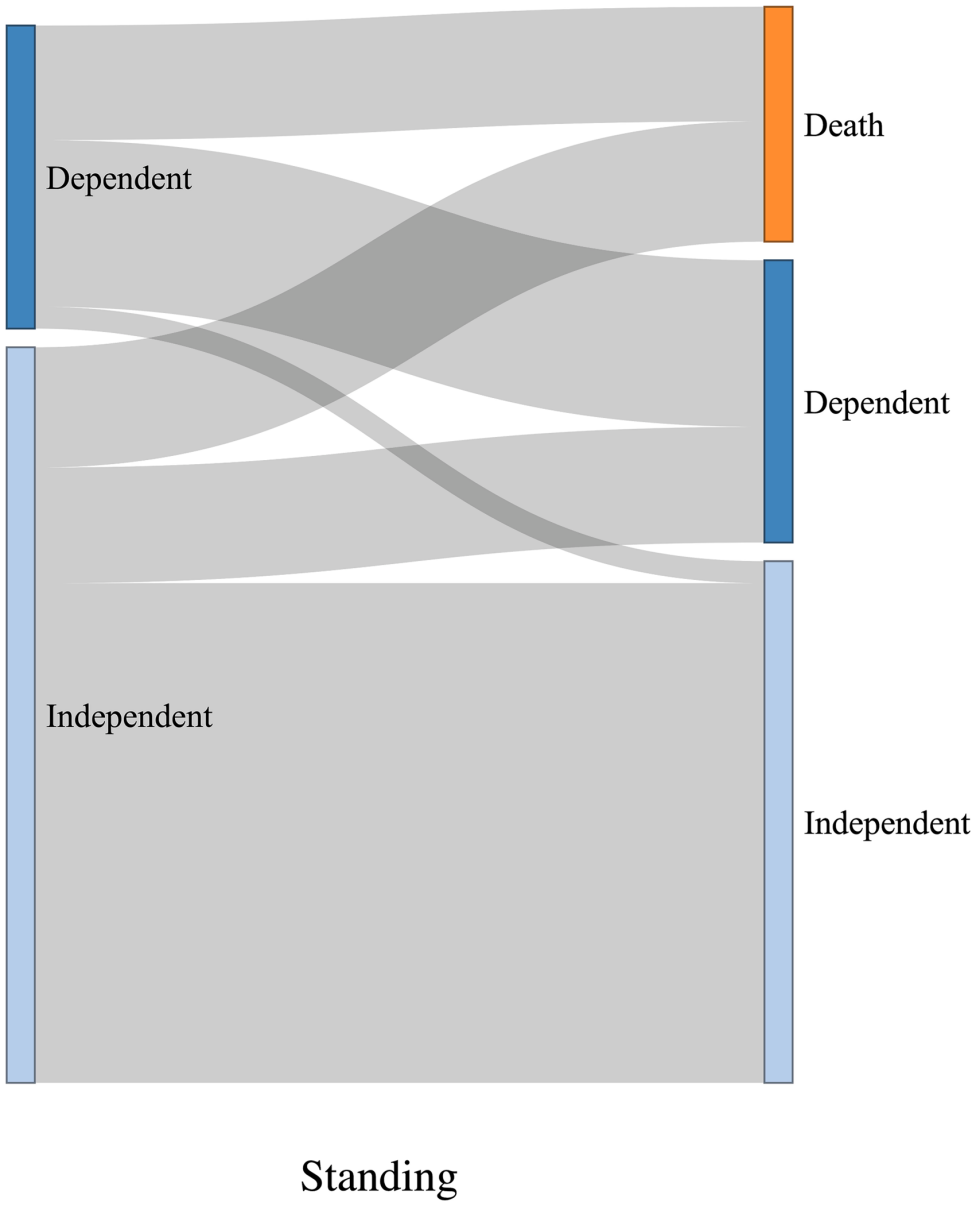
Visualization of change in living status between pre-trauma and 6-month post-trauma for the FSH group. Left side of the diagrams represents pre-trauma, and the right side represents 6-month post-trauma. The height of horizontal columns corresponds to the proportion of patients.

The Sankey diagrams in Figures 9, 10 visualize 6-month outcomes for the two groups and changes in living status. In the SRF group, 62% of those who lived independently returned to independent living after 6 months, while 63% returned to independent living in the FSH group (*p* = 0.8).

## 4 Discussion

In this cohort of TBI patients admitted to the regional neurotrauma center with traumatic findings on head CT, we show that the two most common fall mechanisms leading to TBI were falling from standing height- and stair-related falls, with the latter representing a quarter of all registered falls in OTBIR-N. When comparing the two fall mechanisms, patients in the SRF group were younger and had fewer comorbidities before the injury, but also sustained more severe TBIs. Unadjusted unfavorable outcomes were more common in the FSH patients; however, in the adjusted analysis, a signal of falling down the stairs being associated with unfavorable outcomes was observed, and this likely relates to the adjustment for pre-injury patient factors, including age and comorbidities. SRFs leading to TBI showed a predilection for occurring in the evening and nighttime hours and on the weekends and were more often related to alcohol influence compared to TBI from FSH.

Our results indicate two different patterns of patient and situational factors associated with different fall mechanisms. For FSH, the typical patient can be seen as an elderly patient where patient factors such as comorbidities and frailty may dominate as predisposing factors (2, 23). On the other hand, for SRFs, the typical patient can be seen as a middle-aged person where the situational factors of alcohol influence in the evening and night-time at weekends may dominate in their predisposition. Whether these patterns are unique to this cohort remains undetermined; however, other studies on TBI after stair-related falls show similar trends (12–14).

Overall, the results indicate that both fall mechanisms were associated with an unfavorable functional outcome 6 months after injury in a considerable proportion of patients in the present cohort. With falls representing the leading cause of TBI in an aging population in high-income countries, characterization of fall mechanisms is important to facilitate preventive measures. While reducing patient factors of comorbidity and frailty in the population remains something of a general aim of modern medicine, the situational factors associated with falling down the stairs appear more readily attainable for direct preventive measures. In society, having drinks at home on a Saturday night and walking down the stairs will hardly be regarded as even remotely risky behavior; however, the present results indicate that some falls down the stairs are associated with a non-negligible cost for falling. Middle-aged and older people may consider themselves responsible drinkers, which can make them less likely to recognize the risks (24). As such, public information on the risk associated with alcohol influence and staircases seems warranted in the prevention of such falls.

Some limitations of the present study should be noted. First, it is a single-center study, and this may affect its generalizability per se. Second, it is a study on a cohort of TBI patients from a regional referral center, being the sole provider of neurosurgical services in the region. This implies patient selection in the cohort, as regional patients who would not be considered candidates for neurosurgical intervention may not always be transferred from local hospitals. As such, certain categories of patients, for example, older ages and severely comorbid patients, are likely to be under-represented in the cohort as a whole (25, 26); however, this selection bias should expectantly be similar for both fall mechanisms. Third, the level of detail regarding the available information in the registry on fall mechanisms and contributing factors is limited. This includes data on alcohol levels and drug test results, as well as information on stair ascent/descent, the number of steps, and other person-specific and environmental factors. Consequently, this affects the conclusions that can be drawn. Further studies should collect and analyze data on the conditions at the time of the fall in greater detail.

## 5 Conclusion

SRFs were the second most common fall mechanism leading to TBI after FSH in the present cohort. Compared to FSH, SRFs occurred in younger and less premorbid patients, occurred more often during evening and nighttime hours, and on weekends, were more often associated with alcohol influence, and more commonly resulted in severe TBIs. Public information on the risks associated with the combination of alcohol influence and staircases seems warranted in the prevention of such falls.

## Data Availability

The data analyzed in this study is subject to the following licenses/restrictions: The datasets for this study are not publicly available due to privacy and ethical restrictions. Requests to access these datasets should be directed to uxtvec@ous-hf.no.
